# Lung Vascular Remodeling and Oxidative Damage Induced by Chronic Intermittent Hypoxia

**DOI:** 10.3390/ijms27083434

**Published:** 2026-04-11

**Authors:** Esteban G. Figueroa, Alejandro González-Candia, Alejandro A. Candia, Adolfo A. Paz, Pamela V. Arias, Jorge Rodríguez-Borges, Emilio A. Herrera, Rodrigo L. Castillo

**Affiliations:** 1 Escuela de Obstetricia, Facultad de Ciencias para el Cuidado de la Salud, Universidad San Sebastián, Santiago 8420524, Chile; esteban.figueroa@uss.cl; 2 Laboratory of Fetal Neuroprogramming, Institute of Health Sciences, University of O’Higgins, Rancagua 3655000, Chile; alejandro.gonzalez@uoh.cl; 3 Laboratorio de Función & Reactividad Vascular, Núcleo Interdisciplinario de Fisiología, Biofísica y Fisiopatología, Instituto de Ciencias Biomédicas (ICBM), Facultad de Medicina, Universidad de Chile, Santiago 8380453, Chile; alejandrocandiah@uchile.cl (A.A.C.); adolfo.paz@ug.uchile.cl (A.A.P.); pamearias@ug.uchile.cl (P.V.A.); jrodriguezb@uchile.cl (J.R.-B.); 4 International Center for Andean Studies (INCAS), Universidad de Chile, Putre 1010000, Chile; 5 Departamento de Medicina Interna Oriente, Facultad de Medicina, Universidad de Chile, Providencia, Santiago 7500922, Chile

**Keywords:** chronic intermittent hypobaric hypoxia, lung vascular remodeling, antioxidant enzymes, catalase, thiobarbituric acid reactive substances

## Abstract

High-altitude workers in the Los Andes Mountains, known as “the Chilean miner model,” are exposed to chronic intermittent hypobaric hypoxia (CIHH). This intermittent condition differs from other models of chronic hypoxia, mainly due to the hypoxic pattern and the cardiovascular and pulmonary effects. There are reports of cardiopulmonary dysfunction and remodeling in human and animal models. However, research on some mechanisms of vascular function and the consequences of lung remodeling induced by CIHH is still lacking. Therefore, this study aims to characterize the effects of CIHH exposure on lung structure and redox status in a rat model of the Chilean miner, involving intermittent exposure to chronic cycles of normoxia/hypobaric hypoxia (96 h/96 h) in an experimental hypoxic chamber. Our results demonstrate that CIHH acts as a primary driver of pulmonary vascular remodeling by significantly increasing the medial wall thickness of small pulmonary arteries (<100 μm) and promoting a shift toward a more muscularized phenotype in previously non-muscularized vessels. Structurally, this was characterized by a marked reduction in alveolar space and a significant increase in the thickness of the alveolar-capillary barrier, suggesting impaired gas exchange capacity. These structural changes were strongly associated with a pro-oxidant state, evidenced by increased lipid peroxidation (malondialdehyde levels) and a concomitant reduction in antioxidant enzyme activities, such as superoxide dismutase (SOD) and catalase (CAT), in lung tissue. In conclusion, the CIHH model effectively replicates the complex interplay between chronic oxidative damage and structural lung remodeling, identifying the thickening of the arterial medial wall and alveolar septa as key pathological features of probably CIHH-induced pulmonary hypertension.

## 1. Introduction

Approximately 1–2% of the global population is estimated to reside at altitudes above 2500 m above sea level (masl) [1,2,3], representing a significant global population exposed to hypobaric hypoxia conditions. In this context, it is estimated that between 100,000 and 250,000 workers are exposed annually to periods of acute or chronic intermittent hypobaric hypoxia (CIHH), with Chile, Peru, and Bolivia accounting for approximately 200,000 of these cases [4,5,6]. Over the course of many years, these workers repeatedly ascend to altitudes up to 4800 masl to work in 7–14-day shifts, returning to rest at sea level for equivalent periods of time [7,8,9]. Most of these workers are employed by mining companies. This “miner paradigm” of CIHH [10], unlike continuous hypoxia, acts as a dynamic stimulus that triggers defective adaptive remodeling in the lung bed. The model demonstrates how oxygen alternation promotes morphological changes that decrease the gas exchange area and alter the function of respiratory tissues [11,12]. However, the pulmonary consequences of CIHH, particularly in terms of vascular remodeling and redox-sensitive pathways in the lung, have been poorly characterized in experimental models compared with OSA models [13].

Sustained overproduction of ROS under CIHH conditions induces pro-oxidant imbalance, with associated cardiovascular dysfunction [14,15,16], such as the ROS burst mediated by NADPH oxidase (NOX), cyclooxygenases, and lipoxygenases [17,18,19]. Additionally, the endogenous antioxidant systems—including CAT, superoxide dismutase (SOD), and glutathione peroxidase (GPx)—are activated by the increased ROS generation [17,20,21,22]. Interestingly, four cycles of hypobaric hypoxia enhance antioxidant enzyme expression in the heart [23,24], suggesting an initial period of adaptation and potential cardioprotection. However, the prooxidant effects of CIHH on pulmonary tissue and vessels have not been fully described.

Chronic intermittent hypoxia, such as Obstructive Sleep Apnea, promotes pulmonary arterial remodeling, increasing the inflammatory infiltration and collagen deposition in the lung parenchyma [25,26,27]. This pathophysiological response is based on the activation of transforming growth factor beta (TGF-β) signaling cascades, the increase in hydroxyproline levels, and the production of higher pro-inflammatory cytokines, such as the tumor necrosis factor alpha (TNF-α) and interleukin-6 (IL-6) [26]. The dose–response relationship and temporal progression of these hypoxic effects depend on several factors, including the severity, duration, and pattern of exposure, which finally determine the magnitude and severity of the organ and functional impairment [28,29,30,31]. However, the tissue effects associated with CIHH exposure have not been well characterized.

Despite growing evidence of CIHH-induced adverse effects in various organs and tissues [32], such as the cardiovascular system [33,34], the direct impact on lung cellular architecture and redox homeostasis represents a significant knowledge gap in our understanding of CIHH-mediated organ impairment and long-term functional effects. Therefore, this work aims to characterize the effects of CIHH exposure on pulmonary structure and redox status in a rat model of Chilean miners, exposed to 12 cycles of hypoxia/normoxia.

## 2. Results

### 2.1. CIHH Effects on Weight and Lung Biometry

**Figure 1:**
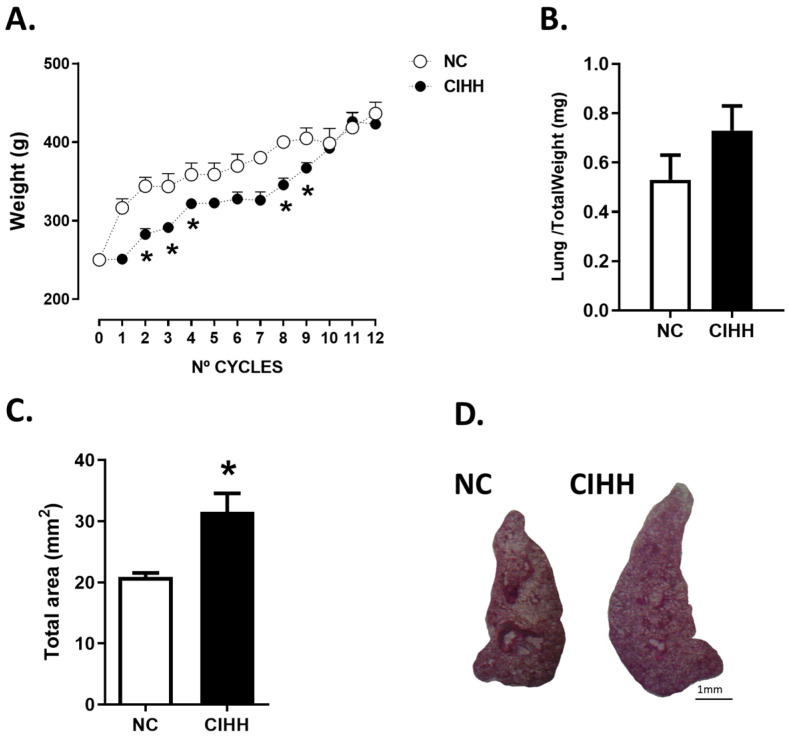
Effects of chronic intermittent hypobaric hypoxia (CIHH) on body weight and lung parenchyma: (**A**) body weight during the experimental protocol, (**B**) lung weight/total weight after the experimental protocol, (**C**) total lung area after the experimental protocol, (**D**) representative image of lung sections, bar scale = 1 mm. The groups are normoxic control (NC, white circles/bars) (*n* = 6) and chronic intermittent hypoxia (CIHH, black circles/bars) (*n* = 6). Values are means ± SEM. Significant differences (*p* ≤ 0.05): * vs. NC.

CIHH exposure induced dynamic changes in body weight throughout the experimental protocol. Rats exposed to CIHH showed a significant decrease in body weight at cycles 2, 3, 4, 8, and 9 compared to the normoxic control (NC) group (Figure 1A).

Although CIHH exposure induced transient changes in body weight during specific cycles, no significant differences in final body weight were observed between groups at the end of the experimental protocol. Accordingly, no differences were observed in the lung-to-body weight ratio (Figure 1B).

Despite the absence of changes in relative lung weight, CIHH significantly increased total lung area compared to NC animals (NC, 20.87 ± 0.64 mm^2^ vs. CIHH, 31.52 ± 3.05 mm^2^; *p* = 0.0159) (Figure 1C,D).

### 2.2. CIHH Effects on Lung Remodeling

**Figure 2:**
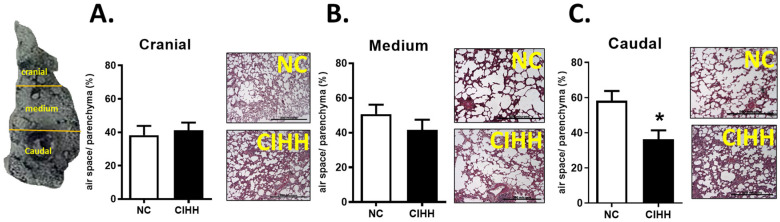
Effects of chronic intermittent hypobaric hypoxia (CIHH) on the lung air space: (**A**) alveolar space/total parenchyma ratio (%) cranial region, (**B**) alveolar space/total parenchyma ratio (%) medium region, (**C**) alveolar space/total parenchyma ratio (%) caudal region. Bar scale in micrographs = 500 μm. The groups are normoxic control (NC, white bars) (*n* = 6) and chronic intermittent hypoxia (CIHH, black bars) (*n* = 6). Values are means ± SEM. Significant differences (*p* ≤ 0.05): * vs. NC.

CIHH exposure induced region-specific alterations in lung parenchyma and vascular structure. The airspace/parenchyma ratio did not differ between groups in the cranial and medial regions; however, a significant reduction was observed in the caudal region of CIHH-exposed rats compared to controls (NC, 58.24 ± 5.52% vs. CIHH, 36.29 ± 5.13%; *p* = 0.0286), indicating localized parenchymal remodeling (Figure 2A–C).

**Figure 3:**
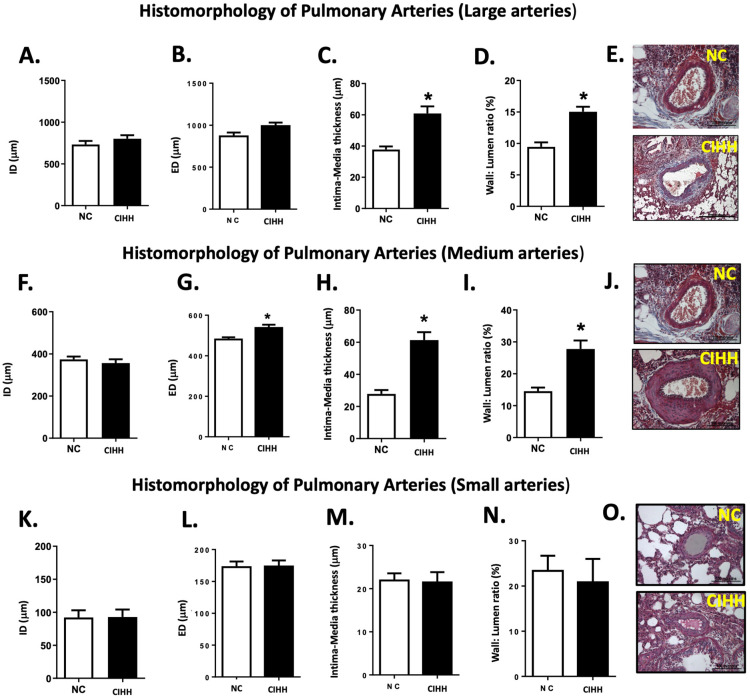
Effects of chronic intermittent hypobaric hypoxia (CIHH) on pulmonary artery morphology. Morphostructural characteristics of large pulmonary arteries (600–1500 μm) vs. (**A**) internal diameter (ID), (**B**) external diameter (ED), (**C**) intima-media thickness, (**D**) wall/lumen ratio (%), and (**E**) representative micrographs (10×) of lung sections with Masson’s trichrome staining. Bar scale in micrographs = 500 μm (CIHH) and 100 μm (NC). Morphostructural characteristics of medium pulmonary arteries (250–600 μm) vs. (**F**) internal diameter (ID), (**G**) external diameter (ED), (**H**) intima-media thickness, (**I**) wall/lumen ratio (%), and (**J**) representative micrographs (40×) of lung sections with Masson’s trichrome staining. Bar scale in micrographs = 100 μm. Morphostructural characteristics of small pulmonary arteries (>250 μm) vs. (**K**) internal diameter (ID), (**L**) external diameter (ED), (**M**) intima-media thickness, (**N**) wall/lumen ratio (%), and (**O**) representative micrographs (40×) of lung sections with Masson’s trichrome staining. Bar scale in micrographs = 100 μm. The groups are normoxic control (NC, white bars) (*n* = 6) and chronic intermittent hypoxia (CIHH, black bars) (*n* = 6). Values are means ± SEM. Significant differences (*p* ≤ 0.05): * vs. NC.

In parallel, CIHH induced marked vascular remodeling that was dependent on arterial caliber. In large pulmonary arteries (600–1500 µm), no differences were observed in internal or external diameter; however, CIHH significantly increased intima-media thickness (NC, 37.71 ± 2.06 µm vs CIHH, 60.83 ± 4.55 µm; *p* < 0.0001) and wall-to-lumen ratio (NC, 9.45 ± 0.72% vs. CIHH, 15.03 ± 0.81%; *p* < 0.0001) (Figure 3A–E).

Similarly, in medium-caliber arteries (250–600 µm), CIHH increased external diameter (NC, 480.2 ± 11.3 µm vs. CIHH, 537.1 ± 16.3 µm; *p* = 0.0345), along with a marked increase in intima-media thickness (NC, 27.83 ± 2.37 µm vs. CIHH, 61.30 ± 4.94 µm; *p* < 0.0001) and wall-to-lumen ratio (NC, 14.54 ± 1.12% vs. CIHH, 27.75 ± 2.68%; *p* < 0.0001) (Figure 3F–J).

In contrast, small pulmonary arteries (<250 µm) did not show significant differences between groups in any morphometric parameter (Figure 3K–O), suggesting that CIHH-induced vascular remodeling predominantly affects medium and large vessels.

**Figure 4:**
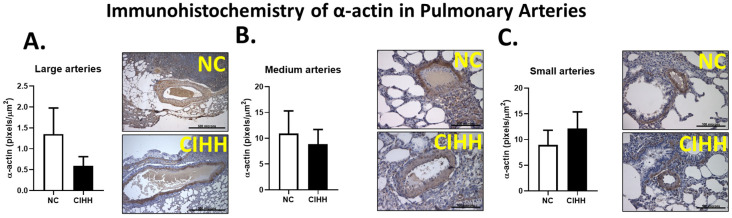
Effects of chronic intermittent hypobaric hypoxia (CIHH) on pulmonary α-actin expression. (**A**) large pulmonary arteries (600–1500 μm) with representative micrographs (40×), (**B**) medium pulmonary arteries (250–600 μm) with representative micrographs (40×), and (**C**) small pulmonary arteries (>250 μm) with representative micrographs (40×). Bar scale in micrographs = 500 μm in (A) and 100 μm in (B,C). The groups are normoxic control (NC, white bars) (*n* = 6) and chronic intermittent hypoxia (CIHH, black bars) (*n* = 6).

Consistent with these findings, α-actin immunoreactivity was similar between NC and CIHH groups across all arterial calibers (Figure 4). Quantification was performed as the ratio of positive pixels normalized to total vessel area, ensuring independence from vessel size. These results indicate that, despite structural remodeling, no changes in smooth muscle contractile markers of expression were detected.

### 2.3. CIHH and HIF Expression in Pulmonary Arteries

**Figure 5:**
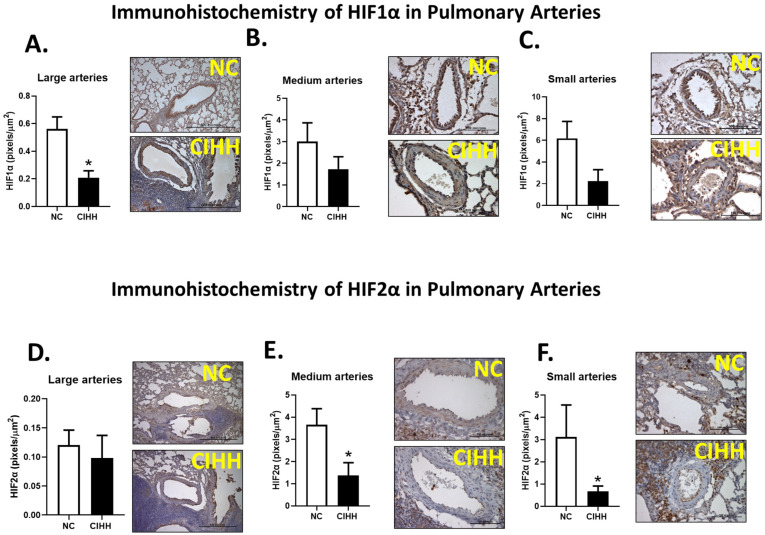
Effects of chronic intermittent hypobaric hypoxia (CIHH) on pulmonary HIF1α and HIF2α expression. HIF1α immunohistochemistry in (**A**) large pulmonary arteries (600–1500 μm) with representative micrographs (40×), (**B**) medium pulmonary arteries (250–600 μm) with representative micrographs (40×), and (**C**) small pulmonary arteries (>250 μm) with representative micrographs (40×). HIF2α immunohistochemistry in (**D**) large pulmonary arteries (600–1500 μm) with representative micrographs (40×), (**E**) medium pulmonary arteries (250–600 μm) with representative micrographs (40×), and (**F**) small pulmonary arteries (>250 μm) with representative micrographs (40×). Bar scale in micrographs = 500 μm in (A,D) and 100 μm in (B,C,E,F). The groups are normoxic control (NC, white bars) (*n* = 6) and chronic intermittent hypoxia (CIHH, black bars) (*n* = 6). Values are means ± SEM. Significant differences (*p* ≤ 0.05): * vs. NC.

CIHH exposure differentially modulated HIF expression depending on arterial caliber. HIF-1α immunoreactivity was significantly reduced in large pulmonary arteries of CIHH-exposed rats compared to NC (NC, 0.562 ± 0.086 vs. CIHH, 0.209 ± 0.049 pixels/µm^2^; *p* = 0.0082) (Figure 5A). In contrast, medium and small arteries showed a decreasing trend that did not reach statistical significance (Figure 5B,C).

Regarding HIF-2α, no differences were observed in large arteries between groups (Figure 5D). However, a significant reduction was detected in medium (NC, 3.660 ± 0.721 vs. CIHH, 1.381 ± 0.571 pixels/µm^2^; *p* = 0.0350) and small arteries (NC, 3.118 ± 1.435 vs. CIHH, 0.669 ± 0.243 pixels/µm^2^; *p* = 0.0221) in the CIHH group compared to controls (Figure 5E,F), indicating a vessel size-dependent effect of CIHH on hypoxia signaling pathways.

### 2.4. CIHH and Antioxidant Enzymes in the Lung

**Figure 6:**
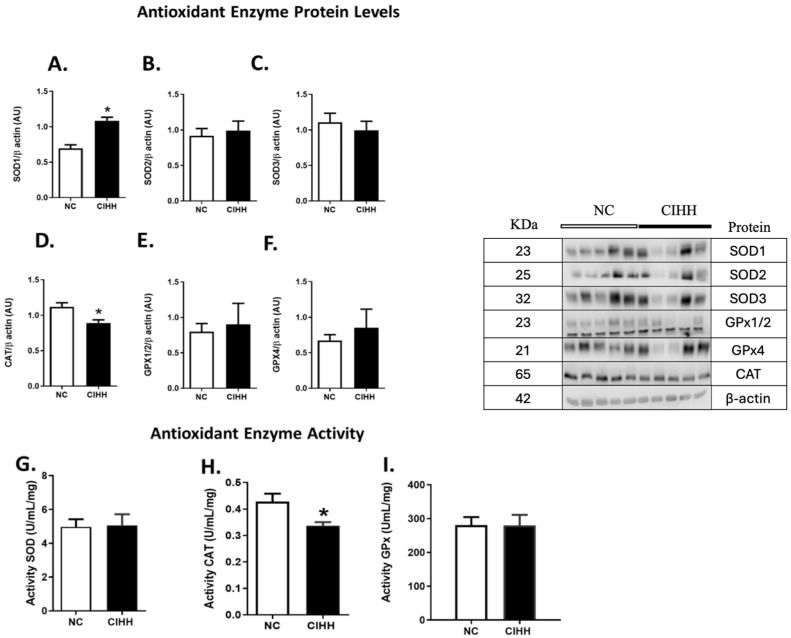
Effects of chronic intermittent hypobaric hypoxia (CIHH) on pulmonary antioxidant enzymes. Lung antioxidant enzyme expression levels: (**A**) SOD1, (**B**) SOD2, (**C**) SOD3, (**D**) CAT, (**E**) GPX1/2, and (**F**) GPX4. Lung antioxidant enzyme activity: (**G**) SODs, (**H**) CAT, (**I**) GPX. Image representative of the Western blot groups. The groups are normoxic control (NC, white bars) and chronic intermittent hypoxia (CIHH, black bars). Values are means ± SEM. Significant differences (*p* ≤ 0.05): * vs. NC.

CIHH exposure induced selective alterations in antioxidant enzyme expression and activity. SOD1 protein levels were significantly increased in the CIHH group compared to NC (NC, 0.35 ± 0.03 AU vs. CIHH, 0.54 ± 0.02 AU; *p* = 0.0079), whereas SOD2, SOD3, GPX1/2, and GPX4 did not show significant differences between groups (Figure 6A–C,E,F).

In contrast, catalase (CAT) expression was significantly reduced in CIHH-exposed animals (NC, 1.12 ± 0.06 AU vs. CIHH, 0.89 ± 0.04 AU; *p* = 0.0317) (Figure 6D). At the functional level, enzymatic activity assays showed no significant changes in SOD and GPX activity (Figure 6G,I), while CAT activity showed a decreasing trend in the CIHH group compared to NC (NC, 0.43 ± 0.03 AU vs. CIHH, 0.34 ± 0.01 AU) (Figure 6H).

These results suggest a selective impairment of the antioxidant defense system, primarily affecting catalase under CIHH conditions.

### 2.5. CIHH and Oxidative Stress in the Lung

**Figure 7:**
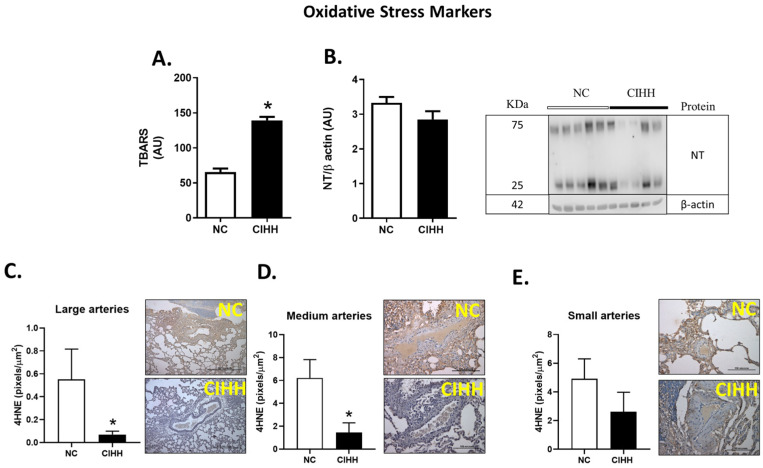
Effects of chronic intermittent hypobaric hypoxia (CIHH) on pulmonary oxidative markers: (**A**) TBARS levels in the lung. (**B**) NT expression levels in the lung. 4HNE immunohistochemistry in (**C**) large pulmonary arteries (600–1500 μm) with representative micrographs (40×), (**D**) medium pulmonary arteries (250–600 μm) with representative micrographs (40×), (**E**) small pulmonary arteries (>250 μm) with representative micrographs (40×). Bar scale in micrographs = 500 μm in (C) and 100 μm in (D,E). Image representative of the Western blot groups. The groups are normoxic control (NC, white bars) and chronic intermittent hypoxia (CIHH, black bars). Values are means ± SEM. Significant differences (*p* ≤ 0.05): * vs. NC.

CIHH exposure resulted in a marked increase in oxidative stress markers in lung tissue. TBARS levels were significantly elevated in CIHH-exposed animals compared to controls (NC, 65.32 ± 5.17 vs. CIHH, 139.3 ± 5.037; *p* = 0.0079), indicating increased lipid peroxidation (Figure 7A).

In contrast, nitrotyrosine (NT) levels did not differ between groups (Figure 7B). However, immunohistochemical analysis of 4HNE revealed a significant reduction in oxidative damage markers in large (NC, 0.552 ± 0.264 vs. CIHH, 0.070 ± 0.029 pixels/µm^2^; *p* = 0.0043) and medium pulmonary arteries (NC, 6.225 ± 1.593 vs. CIHH, 1.448 ± 0.846 pixels/µm^2^; *p* = 0.0303) in the CIHH group (Figure 7C,D), while no differences were observed in small arteries (Figure 7E).

Together, these findings indicate a differential pattern of oxidative stress, with increased lipid peroxidation in lung parenchyma but reduced oxidative markers in specific vascular compartments.

## 3. Discussion

This study demonstrates that CIHH, as a paradigm of occupational exposure in high-altitude Chilean miners, induces a prooxidant redox imbalance and consequently a pulmonary remodeling. Our results suggest that repeated exposure to cycles of hypoxia/normoxia in hypobaric conditions (hypoxic chamber) induces an imbalance between the higher damage by ROS and a drop of cellular antioxidants, leading to tissue damage. Furthermore, pulmonary circulation showed significant morphological changes, suggesting a pivotal contribution of the pulmonary hypertensive/vasoconstrictor component in animals exposed to CIHH. This study represents a significant advance in the understanding of pathophysiological mechanisms that affect pulmonary circulation in repeated cycles of intermittent normobaria/hypobaria in an experimental model.

Our results show that during the protocol, the animals present a decrease in weight in the CIHH group. The drop in total weight was shown in the early stages of CIHH, determining a significant decrease in body weight and energy consumption, as previously described [35,36], and was associated with an increase in energy demand during the hypoxic period [37]. In the CIHH model, previous work about cardiac effects showed a consistent initial decrease in body weight, but no differences in it, nor in energy consumption by the end of the CIHH protocol [10]. In our current protocol, longer exposure time of IHH may reflect greater adrenergic, transcriptional effects on oxygen consumption and tendency toward weight loss [38]. The adrenergic effect explains the central and peripheral mechanisms that determine a high metabolic rate. In our model, we took into account the metabolic rate of a rat, which is four times greater than the metabolic rate of a man [39]. This information was used to calculate the number of days per rat (1 and 2 days) that corresponded approximately to the number of days per man (4 or 8 days) in terms of the effect of exposure to intermittent hypoxia as experienced by Andean workers [40]. Moreover, there were changes in adrenergic tone without alterations in abundance of adrenergic receptors in the heart and vessels [41], determining only plasmatic effects.

Among these mechanisms, increased epinephrine (adrenaline) levels stimulate lipolysis, elevating basal metabolic rate, and promoting the mobilization and oxidation of stored lipids [35,42]. In addition, intermittent hypoxia has been associated with muscle weakness and, in some cases, loss of muscle mass as a result of oxidative stress, even without widespread protein degradation [43,44]. Another relevant mechanism is the alteration of leptin signaling in the carotid bodies, characterized by reduced local protein expression despite normal plasma leptin concentrations. This alteration may contribute to changes in carotid chemoreceptor sensitivity and sustain an anorexigenic effect during chronic hypoxic exposure [45,46].

Concerning the airspace of the lung, CIHH-induced airway and parenchymal remodeling involves a drop of lung function and pro-oxidant changes in the epithelium [47]. In this view, Figure 2, Figure 3, Figure 4 and Figure 5 were designed to comprehensively characterize CIHH-induced pulmonary remodeling, combining (i) changes in parenchyma structure (airspace/parenchyma ratio), (ii) vascular remodeling in arteries of different calibers, and (iii) associations with molecular changes (α-actin expression and hypoxia-inducible factors), allowing a multiscale approach to the phenomenon. CIHH induces structural alterations, such as airway epithelial cell hyperplasia, reticular basement membrane thickening, collagen deposition, parabronchial fibrosis, airway epithelial–mesenchymal transition, and bronchial smooth muscle cell hyperplasia. All these pathophysiologic events have been associated with the decline in lung function and increased airway reactivity [48,49].

Hypoxia is a major mechanism of pulmonary vascular cell proliferation and remodeling, but the severity of the injury depends on the experimental method and the induction of high hypoxic intensity [50,51]. Chronic hypoxia-induced increased pulmonary vascular resistance is determined by active vasoconstriction and remodeling processes [52]. These changes have been shown in different animal models [52,53]. However, in intermittent protocols of hypobaric hypoxia, the results are variable. In fact, our data demonstrate an increased remodeling in the cross-sectional area of the lung and a trend in pulmonary weight in the CIHH group. This may be due to increased pulmonary arterial pressure and increased pulmonary blood volume, inducing a further displacement of pulmonary blood into low-pressure areas, leading to increased pulmonary hydrostatic pressure and pulmonary capillary permeability [54]. Previous work has also suggested that the tachykinin system may be increased in response to hypoxia, leading to enhanced pulmonary vascular albumin extravasation associated with increased expression of the tachykinin receptor (NK-1) [55]. The latter also increases pulmonary edema associated with chronic hypoxia.

Regarding the vascular remodeling, this process is associated with IH exposure, and it has been characterized by an increased muscularization of the peripheral and pulmonary vasculature, with an increase in medial and adventitial thicknesses [56,57]. Additionally, there is adventitia-to-media migration and proliferation and an increase in extracellular matrix deposits along the entire vascular wall [58,59]. Hypoxia may induce proliferation by increasing the production and/or release of different pro-mitogenic molecules such as serotonin, endothelin-1, platelet-derived growth factor (PDGF), and vascular endothelial-derived growth factor (VEGF) [52]. Therefore, CIHH has an outsized remodeling impact in medium-caliber arteries (250–600 um) in the pulmonary bed. However, the pulmonary arterial pressure has not been assessed in our CIHH model. Hence, we propose that the stimulus due to intermittent hypoxic exposure determines a differential activation of vasoconstrictor and remodeling pathways in the pulmonary circulation, being more significant in those arteries with greater flow and shear stress. The increased pressure and shear stress induce eNOS activation and consequently more NO release [60]. However, the pro-oxidant effects on the endothelium determine decreased NO bioavailability [61] and impaired vasodilator function.

Regarding α-actin, no significant changes were observed. This may be explained by the fact that CIHH can induce SMC hyperplasia and hypertrophy while preserving the differentiated contractile phenotype. Hypoxia stimulates the proliferation of a less differentiated SMC subpopulation and increases extracellular matrix deposition, leading to medial thickening, whereas highly differentiated SMCs remain contractile without undergoing phenotypic switching [62]. Bačáková et al. reported that, in VSMCs isolated from pulmonary arteries of rats exposed to chronic hypoxia, α-actin mRNA expression remained unchanged, while other late markers such as calponin and myosin heavy chain increased [63].

Regarding HIF-1α, a significant decrease was observed in large arteries and a slight reduction in medium and small arteries. In contrast, HIF-2α levels remained unchanged in large arteries but decreased in medium and small ones. Under acute hypoxia, HIF-1α is the first to respond, stabilizing within minutes to a few hours after oxygen levels drop. This rapid response triggers early adaptive genes involved in glycolysis and initial angiogenesis [64]. However, HIF-1α accumulation is transient: in endothelial cells under sustained hypoxia, it peaks within the first ~4 h and declines markedly after 8–24 h despite ongoing hypoxic stimulation [64,65]. This decrease reflects a transition from HIF-1 to HIF-2 dominance, driven by metabolic feedback. HIF-1 activation enhances glycolysis and reduces mitochondrial oxygen consumption, reactivating PHDs and promoting its own degradation [66,67,68]. Under intermittent hypoxia, HIF-1α undergoes repeated oscillations—each hypoxic cycle induces a brief stabilization that is truncated by reoxygenation. As the number of cycles increases, the amplitude of HIF-1α induction progressively attenuates. In contrast, HIF-2α exhibits a slower but more sustained response, being predominantly associated with moderate or chronic hypoxia. It regulates long-term adaptive genes, such as those involved in erythropoietin production, antioxidant defense, and vascular remodeling [69,70]. Under intermittent hypoxia, HIF-2α would be expected to progressively stabilize during each 4-day hypoxic period. However, after a short protocol of 12 CIHH cycles, no significant changes were detected in cardiac tissue compared to normoxic controls [36]. The intermittent pattern of hypoxia prevents HIF-2α from remaining continuously elevated, as reoxygenation at the end of each cycle promotes its degradation before it can fully exert its transcriptional effects. Moreover, intermittent hypoxia itself has been shown to hinder HIF-2α accumulation even during the hypoxic phase through mechanisms such as ROS-dependent calpain activation [71]. Indeed, Nanduri et al. (2009) demonstrated that, unlike continuous hypoxia, intermittent exposure markedly decreases HIF-2α levels via calpain-mediated degradation [71].

We found that OS was increased in the lung parenchyma but decreased in small and medium arteries. Oxidative stress occurs when free radicals and other reactive species exceed the available endogenous antioxidants [72]. Reactive oxygen species (ROS), reactive nitrogen species, and their counterpart antioxidant agents are essential for physiological signaling and host defense [73]. However, when imbalances between oxidants and antioxidants occur, they could induce pathological reactions, determining a range of non-respiratory and respiratory diseases [74]. Hypoxic conditions determine an increase in ROS and oxidative stress, particularly at the mitochondrial electron transport chain, the nicotinamide adenine dinucleotide phosphate oxidase (NADPH oxidase), the endoplasmic reticulum, and several enzymes, including uncoupled eNOS and xanthine oxidase (XO) in the lung [75,76]. Oxidative stress by IH has been shown in experimental models such as high altitude, obstructive sleep apnea (OSA), and at sea level [77]. However, our experimental model of chronic intermittent hypobaric hypoxia is based on exposure to 12 cycles of hypobaric hypoxia as a translational model of people working in high-altitude shifts above 2500 masl. Oxidative stress in the lung caused by CIH-derived prooxidant injury is counterbalanced by the activity of the nuclear factor erythroid 2-related factor (Nrf2), a master regulator of cellular redox homeostasis [78]. Upon exposure to oxidants and electrophiles, Nrf2 accumulates in the nucleus, where it binds to antioxidant response elements (ARE) in the upstream regulatory regions of genes encoding detoxification and antioxidant enzymes, leading to their enhanced transcription of GSH production and ROS scavenger compounds [79]. The mechanisms that link the intensity of hypoxia with oxidative damage are multifactorial. Moreover, prooxidant stimulus may be causing a decrease in antioxidant machinery expression and enhanced oxidative stress sources in our CIH model. These findings about tissue redox alterations in other organs were previously published by our group [16].

Although acute intermittent hypoxia is associated with increased ROS production, as mentioned above, the antioxidant defense reports are inconclusive. With respect to CIHH, our study showed no changes in the activity of GPx and SOD in lung tissue in the hypoxic group in comparison with the control group. These findings agree with other models of CIHH [80,81]. On the other hand, the decreased activity of catalase might be associated with lower levels of ROS, which could determine an allosteric regulation in the catalytic subunits of these enzymes [82].

This generates the modification of cofactors like heme-groups that allow the reaction between the enzyme and hydrogen peroxide, decreasing the enzyme activity [83]. Therefore, the redox effects associated with the induction of CIHH and its intermittence can determine the type of tissue response and define whether it is protective or preserves organ function. In this case, our group, using a similar CIHH model, has demonstrated the development of right ventricular hypertrophy, a classical indicator of pulmonary hypertension, supporting the physiological relevance of the vascular remodeling observed in this study [16,72].

Finally, the limitations of this protocol are the lack of functional assessment of the lung, as well as the absence of evaluation of downstream pathways triggered by this type of hypoxia. In addition, tissue collection was performed immediately after the final hypoxic exposure, which does not allow complete discrimination between cumulative chronic adaptations and acute responses to the last hypoxic stimulus. Regarding the potential interplay between increased lung dimensions and altered tissue elasticity, this is indeed a compelling hypothesis; however, since our study did not include lung mechanics (e.g., compliance or resistance) or tensiometric measurements, we cannot draw definitive conclusions on biomechanical properties.

## 4. Materials and Methods

### 4.1. Animals

**Figure 8:**
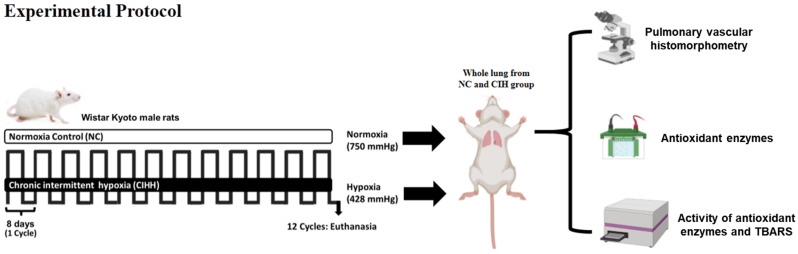
Experimental model. The animals were divided into two experimental groups: the normoxic group was exposed to normobaria (750 Torr, NC), and the chronic intermittent hypoxic group was exposed to hypobaria for 12 cycles (CIHH group) in a hypobaric chamber. Each cycle consisted of 4 days in hypobaria at 430 Torr (equivalent to ~4600 masl) and four days in normobaria at 750 Torr (equivalent to ~560 masl). At the end of the 12 cycles, animals were euthanized immediately, and lung tissue was recovered for analysis of experimental parameters: vascular histomorphotometry, antioxidant enzyme activity, and TBARS levels.

All animal care, maintenance, and procedures were approved by the Bioethics Committee of the Faculty of Medicine, University of Chile (CBA 0865; date approval: 02/09/16, and were carried out under the NIH/NRC Guidelines for the Care and Use of Laboratory Animals. The experiment protocols were performed on 10-week-old Wistar Kyoto male rats (~250 g of weight) from 2016 to 2018. The animals were kept under controlled humidity and temperature conditions (22–25 °C; relative humidity between 45 and 55%, respectively), a 12/12 h light/dark cycle, and free access to feed and drinking water. The animals were divided into 2 experimental groups—chronic intermittent hypobaric hypoxia (CIHH, *n* = 6) and a normoxic control group (NC, *n* = 6)—with two animals per cage. The CIHH was exposed to simulated hypobaria in a hypobaric chamber for 12 cycles. Randomization was not used to assign exposure. Each cycle consisted of 4 days inside the hypobaric chamber, at 428 Torr, equivalent to 4600 masl, and four days in normobaria, ~750 Torr. Control animals (NC group) were maintained under the same environmental and housing conditions as the CIHH group, including temperature (22–25 °C), relative humidity (45–55%), a 12:12 h light/dark cycle, and ad libitum access to food and water. Animals from both groups were housed in pairs (two animals per cage) throughout the experimental protocol (Figure 8) [16]. At the end of the experimental protocol, immediately at the end of the last hypoxic exposure, the animals were euthanized with an anesthetic overdose (sodium thiopental, 150 mg·kg^−1^ IP) for lung tissue extraction. Two animals in the NC group and one in the CIHH group were excluded for not meeting the total exposure time to hypoxia, i.e., the 12 hypoxia/normoxia cycles. The total number, *n* = 6 per group, was used for all experiments in equal form. At the end of the experimental exposure, animals were euthanized immediately after the final hypoxic exposure of the 12th hypoxia/normoxia cycle for lung tissue collection.

### 4.2. Pulmonary Collection

The lungs were removed and weighed. The left lung was divided into two parts; the lower third was quickly frozen and kept at −80 °C for molecular biology assays. The upper 2/3 were fixed in 4% formaldehyde for 24 h (immersion), after which the preservative medium was replaced by phosphate-buffered saline and kept in this medium until the histological technique was performed.

### 4.3. Pulmonary Histology

Fixed lung samples were embedded in paraffin and cut into 5 µm thick slides. Hematoxylin–eosin staining was performed for density in images captured at 100× and 400× with a digital camera coupled to a microscope (Olympus BX-41, New York Microscope Company, Mineola, NY, USA). Histomorphology characteristics of small (<250 μm), medium (250–600 μm), and large (600–1500 μm) pulmonary arteries were studied in the two experimental groups. At the same time, the airspace/parenchyma was studied in 3 pulmonary locations; the lung sectors were determined as cranial, ventral medial, and dorsal medial. Morphological properties (adventitia and muscular thickness; internal and external diameter) were analyzed, and the ratios of wall/lumen were evaluated. Pulmonary arteries were classified according to their caliber, and five arteries per size category were evaluated per animal. Measurements were performed using calibrated images (100 µm scale) in the software Image Pro-Plus 6.2 (Media Cybernetics Inc., Rockville, MD, USA), allowing area-based quantification independent of vessel shape [84].

### 4.4. Antioxidants, Enzymes, and Nitrotyrosine-Related Parameters in the Lung Tissue

Protein levels of catalase (CAT), glutathione peroxidase (GPX)1/2, GPX4, superoxide dismutase (SOD)1, SOD2, SOD3, nitrotyrosine (NT), and β-actin were determined in total lung tissue. The cells were lysed in RIPA buffer (Thermo Scientific, Rockford, IL, USA). Total protein concentration was determined using the Bradford assay. Protein samples were then separated by SDS-PAGE using a 4% stacking gel and a 12% resolving gel under denaturing conditions, followed by transfer onto nitrocellulose membranes. Equal protein loading was verified by Ponceau S staining of each membrane. Each protein was analyzed on a separate membrane using the same protein extracts. The membranes were incubated with primary antibodies (anti-CAT, Abcam, Cambridge, UK, ab1877; anti-GPx-1/2, Santa Cruz Biotechnology, Dallas, TX, USA, sc-74498; anti-GPx-4, Santa Cruz Biotechnology, sc-166570, anti-SOD1, Santa Cruz Biotechnology, sc-17767; anti-SOD2, Millipore, Burlington, MA, USA, 06-984; anti-SOD3, Santa Cruz Biotechnology, sc-376948; anti-NT, Invitrogen, Carlsbad, CA, USA, A-21285; and β-actin, AC-15, Thermo Fisher Scientific, MA1-91399d, respectively), washed and incubated with secondary antibody anti-rabbit or mouse according to the manufacturer instructions.

The signals obtained on immunoblot determinations were scanned and quantified by densitometric analysis using a chemiluminescence detection device (Odyssey Imaging System, Li-Cor Biosciences, Lincoln, NE, USA) [85].

On the other hand, the antioxidant enzyme activities in lung tissue homogenates were measured using commercial assay kits: Superoxide Dismutase (SOD) Activity Assay Kit (706002, Cayman Chemical, Ann Arbor, MI, USA), Catalase (CAT) Activity Assay Kit (707002, Cayman Chemical, Ann Arbor, MI, USA), and Glutathione Peroxidase (GPx) Assay Kit (703102, Cayman Chemical, Ann Arbor, MI, USA), according to the manufacturer’s instructions.

### 4.5. Hypoxia-Inducible Factor and Lipid Peroxidation Immunohistochemistry

Formalin-fixed, paraffin-embedded lung tissue sections were subjected to deparaffinization through sequential immersion in clearing solution (Histo-Clear, #HS2001GLL, National Diagnostics, Atlanta, GA, USA) for three consecutive 5 min intervals, followed by rehydration using graded ethanol series: two immersions in absolute ethanol (5 min each), two treatments in 75% ethanol (5 min each), and a final wash in 50% ethanol (5 min). Subsequently, sections were equilibrated in distilled water for 10 min before proceeding with immunohistochemical analysis. Epitope unmasking was accomplished by heating tissue sections in sodium citrate retrieval buffer (10 mM sodium citrate, 0.05% Tween 20, distilled water, pH 6.0) at 100 °C under pressurized conditions for 40 min, after which sections were allowed to cool in cold water for 20 min to complete the retrieval process.

Tissue sections underwent overnight incubation at 4 °C with specific primary antibodies targeting α-actin (Sigma-Aldrich, St. Louis, MO, USA, A-5228), HIF1α (Novus Biologicals, Centennial, CO, USA, NB100-132), HIF2α (Novus Biologicals, NB100-105), and 4-hydroxynonenal (4HNE) (Abcam Laboratories, ab46545). Antibody binding was visualized using the Mouse/Rabbit PolyDetector Plus DAB HRP Brown detection system (#BSB0261, BioSB, Santa Barbara, CA, USA) following the manufacturer’s specifications. Following DAB substrate development, tissue sections were counterstained with hematoxylin solution (#26754, Electron Microscopy Sciences, Hatfield, PA, USA) for 30–60 s, then subjected to bluing in 1% ammonia solution for 10 s. Final processing involved dehydration through ascending ethanol concentrations and mounting using Entellan mounting medium (#107960, Sigma-Aldrich).

Immunoreactive protein expression was assessed in pulmonary artery intima-media layers through digital quantification of DAB-positive (brown) pixels normalized to total tissue area, utilizing 40× magnification photomicrographs analyzed with Adobe Photoshop CS6 software. A standardized color threshold was applied across all samples to define positive staining, ensuring consistency in signal detection. The same threshold parameters were used for all images, and results were expressed as the ratio of positive pixels relative to total vessel area [86].

### 4.6. Statistical Analyses

All data were expressed as mean ± SEM. The Shapiro–Wilk test was used to evaluate the normality of the data. Accordingly, cardiovascular data, morphometry, and molecular biology assessment were compared statistically by an unpaired *t*-test. Significant differences were determined at a *p*-value ≤ 0.05 (Prism 9.0, GraphPad Software, San Diego, CA, USA).

#### Sample Size Calculation

Regarding sample size, considering differences in tissular TBARS (primary measure) of 65% between groups (NC vs. CIHH), an SD of 0.5, an effect size of 0.5 for the hypobaric hypoxia with 80% power and alpha error of 0.05, 6 rats were needed. (http://www.winepi.net/f108.php, accessed on 28 November 2025). These changes for TBARS levels in the lung were analyzed in previously published research results by our group [23].

## 5. Conclusions

Our animal model representing high-altitude working shifts, such as the pattern implemented in Chilean mining companies and astronomical observatories, among other productive activities, seeks to understand the pulmonary adaptations or lung oxidative parameters in these workers. In our country, where the economy depends on high-altitude activities, it is imperative to explore the effects on the health of such people, or on their related biomarkers, before the organ or functional damage occurs. Furthermore, uncovering the involved pathophysiological mechanisms of pulmonary circulation dysfunction and remodeling in CIHH may offer clues to delineate labor policies and eventual therapeutic and preventive approaches for these workers, such as the pharmacological modulation of antioxidant mechanisms. In this case, the oxidative stress biomarkers can be a non-invasive way to detect susceptibility of subjects to high altitudes and their nocive effects, also complementing personalized health care.

## Data Availability

The original contributions presented in this study are included in the article. Further inquiries can be directed to the corresponding authors.

## Abbreviations

The following abbreviations are used in this manuscript:

CIHH	Chronic Intermittent Hypobaric Hypoxia
IH	Intermittent Hypoxia
IHH	Intermittent Hypobaric Hypoxia
CIH	Chronic Intermittent Hypoxia
NC	Normoxic Control
ROS	Reactive Oxygen Species
NO	Nitric Oxide
NOX	NADPH Oxidase
CAT	Catalase
SOD	Superoxide Dismutase
GPX/GPx	Glutathione Peroxidase
TBARS	Thiobarbituric Acid Reactive Substances
NT	Nitrotyrosine
4HNE	4-Hydroxynonenal
HIF-1α	Hypoxia-Inducible Factor 1 Alpha
HIF-2α	Hypoxia-Inducible Factor 2 Alpha
TGF-β	Transforming Growth Factor Beta
TNF-α	Tumor Necrosis Factor Alpha
IL-6	Interleukin-6
VEGF	Vascular Endothelial Growth Factor
PDGF	Platelet-Derived Growth Factor
eNOS	Endothelial Nitric Oxide Synthase
OSA	Obstructive Sleep Apnea
VSMC	Vascular Smooth Muscle Cell
ARE	Antioxidant Response Element
Nrf2	Nuclear Factor Erythroid 2-Related Factor 2
masl	Meters Above Sea Level
SEM	Standard Error of the Mean
SDS-PAGE	Sodium Dodecyl Sulfate–Polyacrylamide Gel Electrophoresis
DAB	3,3′-Diaminobenzidine
